# Draft Genome Sequence of the Bacterium *Cupriavidus* sp. Strain D39, Inhabiting the Rhizosphere of Pea Plants (Pisum sativum L.)

**DOI:** 10.1128/mra.01354-22

**Published:** 2023-03-21

**Authors:** Polina Guro, Pavel Ulianich, Alexander Shaposhnikov, Oleg Yuzikhin, Denis Karlov, Anna Sazanova, Vera Safronova, Andrey Belimov

**Affiliations:** a All-Russia Research Institute for Agricultural Microbiology (ARRIAM), St. Petersburg, Russian Federation; Wellesley College

## Abstract

We report the draft genome sequence of *Cupriavidus* sp. strain D39, associated with the roots of pea plants. The genome is characterized by a GC content of 63.62% and a total length of 7.7 Mbp and contains several putative genes associated with resistance to metals and plant growth promotion.

## ANNOUNCEMENT

Plants growing in acidic soils suffer from elevated concentrations of mobile aluminum ions (1). An essential feature of the species Cupriavidus basilensis is its ability to resist toxic metals (2–4) and degrade xenobiotics (5–7).

*Cupriavidus* sp. strain D39 was isolated from the fresh (washed and homogenized using a sterile mortar and pestle) roots of pea plants (Pisum sativum L.) line SGE grown in an acid Haplic Leptosol (pH_KCl_, 4.5; collected at 59.449479°N, 33.818877°E) by the standard serial dilution method using agar growth medium (2 g L^−1^ mannitol, 2 g L^−1^ fructose, 1 g L^−1^ sodium citrate, 0.5 g L^−1^ yeast extract, 1 g L^−1^ KH_2_PO_4_, 0.25 g L^−1^ NH_4_NO_3_, 0.25 g L^−1^ MgSO_4_, 20 g L^−1^ agar; pH 5.6) supplemented with 130 mg L^−1^ AlCl_3_. The details of the isolation procedures were described previously (8). The same medium was used for obtaining and storing the pure culture at −80°C and preparing separate colonies for further study. A single colony of the frozen stock was inoculated into Reasoner’s 2A (R2A) medium broth and incubated for 3 days at 28°C. Then, genomic DNA was extracted using a DNeasy blood and tissue kit (Qiagen, Germany) according to the manufacturer’s recommendations, omitting the DNA shearing step. Library preparation was performed using the Oxford Nanopore Technologies ligation sequencing kit (SQK-LSK109; ONT, UK) and the native barcoding and expansion kits (EXP-NBD104 and EXP-NBD114; ONT) with the isolated DNA without size selection. The whole genome was sequenced with long reads using a MinION sequencer with a flow cell (R.9.4.1; ONT). For this genome, 31,827 raw reads and 188.5 million bases were generated, with an average length of 5,922 bp. The reads were base called and demultiplexed and the barcodes were trimmed using the program Guppy v.5.0.11 (9). Assembly was conducted using Flye v.2.9, with polishing repeated twice: “-i 2” (10). The assembly was then circularized using Circlator v.1.5.5, with the following parameters recommended for ONT assembly, without rotation: “–merge_min_id 85 –merge_breaklen 1000” (11). Default parameters were used for all software unless otherwise noted.

Sequencing generated 11 contigs with a total genome size of 7,740,597 bp and a 44-fold average coverage. The quality of the assembly was evaluated using QUAST v.5.0.2; the GC content was 63.6%, and the *N*_50_ value was 5,336,522 bp (12). The assembly was annotated using NCBI’s PGAP v.6.3 software (13). The draft genome contains 7,594 coding sequences and 76 RNA genes. Taxonomic identification was based on the average nucleotide identity (ANI) and by BLASTn comparison of the 16S rRNA gene between the isolate and the type strain *C. basilensis* DSM 11853 (GenBank accession number GCA_008801925.2). The ANI comparison showed identity values of 93.19% based on MUMmer (ANIm) and 88.35% based on BLAST (ANIb), using the JSpeciesWS v.2.7.0 server (accessed 12 November 2022) (14); in addition, rRNA genes were predicted using Barrnap v.0.9 (https://github.com/tseemann/barrnap). The 16S rRNA gene sequence of strain D39 was closely related (identity, 99.74%) to that of the type strain *C. basilensis* DSM 11853.

The genome of *Cupriavidus* sp. D39 includes various putative proteins that have functions related to stress and metal tolerance, including bacterial siderophores and hemoglobins, in addition to genes that are predicted to be involved in plant growth promotion, such as IAA (indole-3-acetic acid) biosynthesis and ACC (1-aminocyclopropane-1-carboxylic acid) deaminase.

### Data availability.

All data are available at GenBank under BioProject accession number PRJNA895216. The BioSample accession number is SAMN31497846. The raw MinION data can be found under SRA accession number SRR22426105. The GenBank assembly accession number is JAPNLE010000000.
